# Early-Life Factors and Body Mass Index Trajectories Among Children in the ECHO Cohort

**DOI:** 10.1001/jamanetworkopen.2025.11835

**Published:** 2025-05-22

**Authors:** Chang Liu, Sy-Miin Chow, Izzuddin M. Aris, Dana Dabelea, Jenae M. Neiderhiser, Leslie D. Leve, Clancy Blair, Diane J. Catellier, Lance Couzens, Joseph M. Braun, Assiamira Ferrara, Judy L. Aschner, Sean C. L. Deoni, Anne L. Dunlop, James E. Gern, Katherine Rivera-Spoljaric, Tina V. Hartert, Gurjit K. Khurana Hershey, Margaret R. Karagas, Elizabeth M. Kennedy, Catherine J. Karr, Emily S. Barrett, Qi Zhao, Barry M. Lester, Jennifer F. Check, Jennifer B. Helderman, Thomas G. O’Connor, Jerod M. Rasmussen, Joseph B. Stanford, Nicole L. Mihalopoulos, Rosalind J. Wright, Robert O. Wright, Kecia N. Carroll, Cindy T. McEvoy, Carrie V. Breton, Leonardo Trasande, Scott T. Weiss, Amy J. Elliott, Christine W. Hockett, Jody M. Ganiban

**Affiliations:** 1 Department of Psychology, Washington State University, Pullman; 2 Department of Human Development and Family Studies, The Pennsylvania State University, University Park; 3 Department of Population Medicine, Harvard Medical School and Harvard Pilgrim Health Care Institute, Boston, Massachusetts; 4 Department of Epidemiology, Colorado School of Public Health, University of Colorado Anschutz Medical Campus, Aurora; 5 Lifecourse Epidemiology of Adiposity and Diabetes (LEAD) Center, University of Colorado Anschutz Medical Campus, Aurora; 6 Department of Pediatrics, University of Colorado Anschutz Medical Campus, Aurora; 7 Department of Psychology, The Pennsylvania State University, University Park; 8 College of Education, University of Oregon, Eugene; 9 Department of Applied Psychology, New York University, New York; 10 RTI International, Research Triangle Park, North Carolina; 11 Department of Epidemiology, Brown University, Providence, Rhode Island; 12 Kaiser Permanente Northern California Division of Research, Oakland; 13 Upstream Prevention of Adiposity and Diabetes Mellitus (UPSTREAM) Center, Kaiser Permanente Northern California Division of Research, Oakland; 14 Center for Discovery and Innovation, Department of Pediatrics, Hackensack Meridian School of Medicine, Nutley, New Jersey; 15 Department of Pediatrics, Albert Einstein College of Medicine, Bronx, New York; 16 Department of Pediatrics, Brown University, Providence, Rhode Island; 17 Department of Gynecology and Obstetrics, Emory University School of Medicine, Atlanta, Georgia; 18 Department of Pediatrics, University of Wisconsin–Madison, Madison; 19 Department of Pediatrics, Washington University in St. Louis, St. Louis, Missouri; 20 Department of Medicine, Vanderbilt University, Nashville, Tennessee; 21 Division of Asthma Research, Department of Pediatrics, Cincinnati Children’s Hospital Medical Center, Cincinnati, Ohio; 22 Department of Epidemiology, Dartmouth College, Hanover, New Hampshire; 23 Department of Environmental & Occupational Health Sciences, University of Washington, Seattle; 24 Department of Pediatrics, University of Washington, Seattle; 25 Department of Biostatistics and Epidemiology, Rutgers School of Public Health, Rutgers, The State University of New Jersey, Piscataway; 26 Environmental and Occupational Health Sciences Institute, Rutgers, The State University of New Jersey, Piscataway; 27 Department of Preventive Medicine, The University of Tennessee Health Science Center, Memphis; 28 Women and Infants Hospital Rhode Island, Providence; 29 Wake Forest University School of Medicine, Winston Salem, North Carolina; 30 Department of Psychiatry, University of Rochester Medical Center, Rochester, New York; 31 Department of Neuroscience, University of Rochester Medical Center, Rochester, New York; 32 Department of Obstetrics and Gynecology, University of Rochester Medical Center, Rochester, New York; 33 Department of Pediatrics, University of California, Irvine; 34 Department of Family & Preventive Medicine, The University of Utah, Salt Lake City; 35 Department of Pediatrics, The University of Utah, Salt Lake City; 36 Department of Public Health, Icahn School of Medicine at Mount Sinai, New York, New York; 37 Department of Environmental Medicine and Climate Science, Icahn School of Medicine at Mount Sinai, New York, New York; 38 Department of Pediatrics, Icahn School of Medicine at Mount Sinai, New York, New York; 39 Department of Pediatrics, Papé Family Pediatric Research Institute, Oregon Health & Science University, Portland; 40 Population and Public Health Sciences, Keck School of Medicine, University of Southern California, Los Angeles; 41 Department of Pediatrics, New York University Grossman School of Medicine, New York; 42 Department of Population Health, New York University Grossman School of Medicine, New York; 43 New York University Wagner Graduate School of Public Service, New York; 44 Channing Division of Network Medicine, Department of Medicine, Brigham and Women’s Hospital and Harvard Medical School, Boston, Massachusetts; 45 Avera Research Institute, Sioux Falls, South Dakota; 46 Department of Pediatrics, University of South Dakota School of Medicine, Sioux Falls; 47 Department of Psychological & Brain Sciences, Columbian College of Arts & Sciences, The George Washington University, Washington, DC

## Abstract

**Importance:**

Identifying atypical body mass index (BMI) trajectories in children and understanding associated, modifiable early-life factors may help prevent childhood obesity.

**Objective:**

To characterize multiphase BMI trajectories in children and identify associated modifiable early-life factors.

**Design, Setting, and Participants:**

This cohort study included longitudinal data obtained from January 1997 to June 2024, from the Environmental influences on Child Health Outcomes (ECHO) cohort, which included children aged 1 to 9 years with 4 or more weight and height assessments. Analyses were conducted from January to June 2024.

**Exposures:**

Prenatal exposure to substances and stress (smoking, alcohol, depression, anxiety), maternal characteristics (prepregnancy BMI, gestational weight gain), child characteristics (preterm birth, birth weight, breastfeeding), and demographic covariates.

**Main Outcomes and Measures:**

BMI (calculated as weight in kilograms divided by length in meters squared for children aged 1 and 2 years and as weight in kilograms divided by height in meters squared for children older than 2 years) obtained using medical records, staff measurements, caregiver reports, or remote study measures. The analysis was conducted using a multiphase latent growth mixture model.

**Results:**

This study included 9483 children (4925 boys [51.9%]). Two distinct 2-phase BMI patterns were identified: typical and atypical. The typical group (n = 8477 [89.4%]) showed linear decreases in BMI (*b*_2_, −0.23 [95% CI, −0.24 to −0.22]), with the lowest BMI at age 6 years (95% CI, 5.94-6.11), followed by linear increases from 6 to 9 years (slope difference [*b*_4_ − *b*_2_], 0.81 [95% CI, 0.76-0.86]; mean BMI at 9 years: 17.33). The atypical group (n = 1006 [10.6%]) showed a stable BMI from ages 1 to 3.5 years (*b*_6_, 0.06 [95% CI, −0.04 to 0.15]), followed by rapid linear increases from ages 3.5 to 9 years (slope difference [*b*_8_ − *b*_6_], 1.44 [95% CI, 1.34-1.55]). At age 9 years, this group reached a mean BMI (26.2) that exceeded the 99th percentile. Prenatal smoking, high prepregnancy BMI, high gestational weight gain, and high birth weight were key risk factors for the atypical trajectory.

**Conclusions and Relevance:**

In this cohort study of children in the ECHO cohort, analyses identified children on the path to obesity as early as age 3.5 years. Modifiable factors could be targeted for early prevention and intervention programs aimed at reducing childhood obesity.

## Key Points

**Question:**

How are early-life factors associated with body mass index (BMI) trajectories in children?

**Findings:**

In this cohort study of 9483 children, there were 2 distinct BMI trajectories: a typical trajectory (89% of children) and an atypical trajectory with an earlier BMI increase (11% of children). Prenatal smoking, high maternal prepregnancy BMI, high gestational weight gain, and high birth weight were associated with the atypical trajectory.

**Meaning:**

The findings of this study may help identify an atypical BMI trajectory during early childhood and associated risk factors, indicating potential opportunities for preventing childhood obesity.

## Teaser

This cohort study assesses whether early-life factors are associated with body mass index (BMI) trajectories in children from the Environmental influences on Child Health Outcomes (ECHO) cohort.

## Introduction

Childhood obesity, defined as a body mass index (BMI) at or above the 95th percentile for age and sex due to excessive body fat, is a major risk factor for chronic health complications in children.^1,2^ Without intervention, children exhibiting high BMI trajectories during childhood are more likely to develop overweight or obesity as adolescents and adults and are at higher risk for a range of metabolic and cardiovascular diseases throughout their lifetimes.^3,4^ Therefore, it is critical to examine developmental pathways that may lead to unhealthy vs healthy BMIs during childhood and to identify modifiable early-life factors that are associated with BMI trajectories.

We used a multiphase growth mixture model^5,6^ to capture critical qualitative shifts in BMI developments during childhood that are best regarded as abrupt shifts and distinct phases as opposed to quantitative changes that unfold smoothly over time. These phases differ in the pace and direction of change and variations in the timing of phase transitions.^7,8,9^ BMI rapidly increases during the first year of life, reaching its peak around 1 year of age (phase 0). From age 1 year onward, BMI typically declines during early childhood (phase 1), reaching a nadir between ages 5 and 7 years, followed by increasing BMI (ie, adiposity rebound [phase 2]).^10^ Due to limited intensive longitudinal assessments during phase 0, our study focuses on phases 1 and 2. Phases 1 and 2 are connected by a change point (ie, the nadir), which heralds a change in the direction and rate of BMI growth.^11,12^ Previous work indicates that the timing of the change point and the growth rate during the adiposity rebound have significant health implications. An earlier age at adiposity rebound is a biological marker of accelerated growth and a risk factor for the emergence of obesity during childhood and cardiometabolic risk in adolescence.^12,13,14,15^ Moreover, more rapid BMI gains during the adiposity-rebound phase may indicate higher risk of later obesity.^16^ Notably, some children bypass the normative decline phase and show continuous rapid BMI increases throughout childhood; these children may be at the highest risk for obesity.^17,18,19,20^

A key limitation of most previous research is the inability to assess within-person transitions through theoretically informed developmental phases and individual differences (eg, atypicality) in transition patterns and timing (eg, early transition, continuous increases without normative decline). This includes studies using mixed-effects models with polynomial functions or nonlinear splines,^17,21^ traditional growth mixture modeling,^18,22^ or cluster analyses,^23,24^ which overlook important variations that may be clinically significant. To address these limitations, we applied a novel multiphase latent growth mixture modeling approach to characterize complex multiphase trajectories in child BMI from toddlerhood to preadolescence, the timing of phase transitions, and deviations from typical trajectories that unfold in different phases. We also examined whether modifiable early-life factors, including prenatal exposures, and maternal and child characteristics were associated with the timing of phase transitions, growth rates within phases, and typical vs atypical trajectories after controlling for demographics.

The selection of the early-life factors and covariates was guided by the developmental origins of the health and disease (DOHaD) framework,^25^ which emphasizes that exposures during prenatal and early postnatal life may have long-lasting effects on child BMI trajectories. For example, prenatal exposure to substances and stress may alter fetal metabolism and adipose tissue development, showing lasting effects on BMI development.^26,27^ Maternal prepregnancy BMI and gestational weight gain may reflect both genetic influences and the prenatal nutritional environment, which may shape fetal growth patterns and subsequent development.^28,29^ Child characteristics, including preterm birth, birth weight, and early feeding practices, represent early postnatal factors that may influence BMI trajectories.^30,31,32^ Moreover, demographic factors, such as child sex, race and ethnicity, and maternal educational level, were included as covariates, given well-documented disparities in childhood obesity rates across these social determinants of health.^33,34^

Based on previous research, we hypothesized that we would identify 2 distinct BMI trajectory patterns: a typical trajectory showing the normative BMI decline followed by adiposity rebound and an atypical trajectory showing early and rapid BMI increases. We further hypothesized that modifiable early-life factors (eg, prenatal smoking, high maternal prepregnancy BMI, excessive gestational weight gain, high birth weight, and nonbreastfeeding) would be associated with increased risk of following the atypical BMI trajectory, more rapid BMI growths, and earlier timing of BMI increase.

## Methods

### Participants

Study participants were from the Environmental influences on Child Health Outcomes (ECHO) cohort, including 69 longitudinal pediatric cohorts from 44 US states and Puerto Rico, with over 57 000 children from diverse racial and ethnic, geographical, and socioeconomic backgrounds.^35,36^ In this cohort study that included data obtained from January 1997 to June 2024, we included children with 4 or more BMI assessments from ages 1 to 9 years. This resulted in the inclusion of 9483 children born between 1997 and 2019 from 23 cohorts. Demographic information for the analysis sample is provided in eTable 1 in Supplement 1. Ethical approval was obtained from each cohort study site’s institutional review board. Primary caregivers provided written informed consent, and child assent where appropriate, before participating. This study followed the Strengthening the Reporting of Observational Studies in Epidemiology (STROBE) reporting guideline for cohort studies by addressing all 22 required items in the checklist.

### Outcome: Child BMI

Child weight and height data were obtained from medical records (58.1%), staff measurements (37.7%), caregiver reports (3.9%), or remote study measures or child self-report (0.3%) between ages 1 and 9 years. BMI was calculated as weight in kilograms divided by length in meters squared for children aged 1 and 2 years and as weight in kilograms divided by height in meters squared for children older than 2 years. We focused on BMI trajectories starting from age 1 year rather than earlier because BMI trajectories during the first year of life are characterized by rapid increases (phase 0) that follow distinct patterns from later growth. For each age point, we created 1-year age bins centered at whole years (eg, for age 2 years, we included assessments from ages 1.5 to 2.5 years), except for age 1 year, in which we used a 6-month bin (ages 1.0 to 1.5 years) to avoid overlap with the rapid growth phase in infancy. When multiple BMI assessments were available within an age bin, we calculated the mean to create 1 aggregate score per age bin per participant. After this aggregation, participants contributed a mean (SD) of 5.60 (1.57) BMI assessments, with a range of 4 to 9 assessments per participant, and the sample included 53 152 total BMI observations.

### Exposures

Prenatal exposures to substances and stress assessed by maternal self-report and/or medical records included prenatal smoking (yes vs no), prenatal alcohol use (yes vs no), and prenatal or perinatal depression and/or anxiety diagnosis (yes vs no). Maternal characteristics were obtained using maternal self-report, staff measurements, and/or medical records, including prepregnancy BMI (between 12 months’ preconception and the end of the first trimester) and gestational weight gain (categorized as less than recommended, met the recommendation, and gained more than recommended per the 2009 Institute of Medicine recommendation).^37^ We obtained child characteristics through caregiver reports and/or medical records, which included preterm status (gestational age <37 weeks), birth weight (in kilograms), and breastfeeding status (ever breastfed vs not). eTable 2 in Supplement 1 provides additional details about our assessment methods.

### Covariates

We obtained the following demographic covariates using caregiver reports: child biological sex (boy or girl), child race and ethnicity, and maternal educational level during pregnancy (less than high school; high school degree, General Educational Development, or equivalent; some college; bachelor’s degree; professional or doctorate degree). Child race and ethnicity were reported by caregivers, with options defined by investigators. We classified children’s race and ethnicity into Hispanic or Latino, non-Hispanic Black (hereinafter Black), non-Hispanic White (hereinafter White), and non-Hispanic other (hereinafter other [including American Indian or Alaska Native, Asian, Native Hawaiian or Other Pacific Islander, and more than 1 race]). We were interested in examining child race and ethnicity given the well-documented disparities in obesity rates across different racial and ethnic groups.^33,34^ Child race and ethnicity are considered social constructs that may expose children to different lived experiences, environmental exposures, and structural inequities, shaping BMI trajectories during childhood.^38,39^

### Statistical Analysis

Analyses were conducted from January to June 2024. We constructed a multiphase latent growth mixture model^6,40,41^ to characterize child BMI trajectories in several steps using Mplus, version 8.4 (Muthén and Muthén).^42^ First, we built a baseline single-phase latent growth mixture model to identify the number of distinct growth patterns (ie, latent classes) within the sample before expanding to the multiphase model. Single-phase growth models conceptualize development as a single phase, focusing on specification and extraction of patterns of change that span the entire study time span. Therefore, this analysis provides evidence of distinct growth patterns across classes (latent or unobserved subgroups) of individuals but does not characterize within-individual changes in growth features (ie, direction and rate of growth) across phases, nor does it allow for the examination of the presence and determinants of change points. Akaike information criterion (AIC), Bayesian information criterion (BIC), and sample size (>5% in each group) were used to compare the model fit of single-phase latent growth models with 1, 2, and 3 classes (latent subgroups).^43^

In the second step, we expanded the best-fitting single-phase model to investigate the need to include additional phases for different latent classes. For example, starting with a preferred 2-class model (selected based on information criterion measures and sample size), we proceeded to determine, for each class, the number of distinct phases of growth in BMI, characteristics of the growth trajectories within each phase, the time location of the change point (ie, the point of transitioning from one phase to the next), and possible determinants (eg, person-specific risk and protective factors) of these growth and change point parameters. For reasons of parsimony and due to the relatively limited number of measurement points available from all participants, we adopted the bilinear (or piecewise-linear) approach, which is widely used in growth-curve literature,^5,41^ and limited our consideration of growth trajectories within each phase to be linear, with any nonlinearity in growth (eg, decelerating growth in BMI) captured as transitions through phases of linear growth with distinct slopes. Compared with the single-phase models, multiphase models defined class membership (C) based on a broader set of between-individual differences in growth patterns that unfolded either in phase 1 or phase 2, including the initial BMI level (intercept) and rate of change (slope) in phase 1; the change point (eg, the age at which the BMI trajectory shifted direction); and the slope change from phase 2 to phase 1 due to model specification (slope differences between phase 2 and phase 1). Detailed model specification is provided in eMethods 1 in Supplement 1.

In the last set of analyses, we examined early-life factors of BMI trajectories using a sequential modeling approach. First, we included only demographic covariates (child sex, race and ethnicity, and maternal educational level) to examine their associations with class membership (odds ratio [OR]), the change point, and growth rates within each phase. Next, we examined 3 sets of early-life factors separately, each time controlling for demographic covariates, including prenatal exposures, maternal characteristics, and child characteristics. Finally, we conducted a full model including all variables simultaneously. Additionally, to examine potential sex differences in BMI trajectories, we conducted separate analyses for boys and girls following the same analytical steps described above.

Consistent with previous research, ^44,45^ we used the full information maximum likelihood (FIML) estimation to handle missing BMI assessments in the multiphase latent growth mixture model. We used multiple imputation with chained equations (MICE)^46^ to address missing data in exposure variables and covariates. Detailed information is provided in eMethods 2 in Supplement 1.

## Results

This study included 9483 children (4925 boys [51.9%] and 4558 girls [48.1%]). Of the total participants, 1762 (18.6%) were Black, 1854 (19.6%) were Hispanic or Latino, 4358 (46.0%) were White, 1318 (13.8%) were of other race or ethnicity, and 191 (2.0%) were missing race or ethnicity data. Descriptive statistics (mean, SD, missing rate) for child BMI at each age and exposure variables are provided in Table 1.

**Table 1.** Descriptive Statistics for Child BMI by Age and Exposure Variables (N = 9483)

Variable	Values	Missing data^a^
Child BMI, mean (SD)		
Age, y		
1	16.97 (0.02)	2379 (25.1)
2	16.62 (0.02)	937 (9.9)
3	16.40 (0.02)	1414 (14.9)
4	16.22 (0.02)	2140 (22.6)
5	16.31 (0.03)	2837 (29.9)
6	16.55 (0.04)	4712 (49.7)
7	17.03 (0.04)	4850 (51.1)
8	17.80 (0.06)	6004 (63.3)
9	18.67 (0.08)	6924 (73.0)
Prenatal exposure, No (%)		
Smoking		
Yes	917 (9.7)	996 (10.5)
No	7570 (79.8)	
Alcohol use		
Yes	1726 (18.2)	1034 (10.9)
No	6723 (70.9)	
Depression diagnosis^b^		
Yes	1050 (11.1)	3908 (41.2)
No	4525 (47.7)	
Anxiety diagnosis^b^		
Yes	490 (5.2)	4952 (52.2)
No	4041 (42.6)	
Maternal characteristics		
Prepregnancy BMI, mean (SD)	27.44 (0.08)	1777 (18.7)
Gestational weight gain, No (%)		
Gained less than recommended	1354 (14.3)	2396 (25.3)
Met the recommendation	2013 (21.2)	
Gained more than recommended	3720 (39.2)	
Child characteristics		
Breastfeeding, No (%)		
Ever breastfed	6990 (73.7)	1545 (16.3)
No	948 (10.0)	
Preterm status, No (%)		
Preterm	1164 (12.3)	113 (1.2)
Full term	8206 (86.5)	
Birth weight, mean (SD), kg	3.21 (0.01)	76 (0.8)

Abbreviation: BMI, body mass index (calculated as weight in kilograms divided by length in meters squared for children aged 1 and 2 years and as weight in kilograms divided by height in meters squared for children older than 2 years).

^a^ All data in this column are presented as the No. (%) of missing participants.

^b^ Includes perinatal exposure.

Note that all coefficients presented herein were unstandardized. Therefore, effects on change points were interpreted in years, and associations with BMI measures were interpreted in BMI units.

### Identification of Growth Patterns

#### Single*-*Phase Latent Growth Mixture Model

The AIC and BIC decreased from the 1-class model to the 3-class model, indicating improved fit with each additional class (eTable 3 in Supplement 1 provides detailed model fit statistics comparisons). However, because of the small proportion of the first group (2.6%) in the 3-class model and for parsimony, a 2-class model was selected as the optimal model for the multiphase analyses (eFigure in Supplement 1).

#### Multiphase Latent Growth Mixture Model

Based on the single-phase model results, we constructed a 2-class, 2-phase linear spline model. The average posterior probability of membership for each group ranged from 0.91 to 0.98, and the entropy was 0.88, indicating satisfactory model fit and distinct classes. As shown in the Figure, the 8477 children in the typical group (89.4% of the sample) had a mean BMI of 16.72 (*b*_1_, 95% CI, 16.69-16.76) at age 1 year, which is approximately the 50th percentile for both boys and girls. Subsequently, these children, on average, showed estimated linear BMI decreases from ages 1 to 6 years (phase 1: *b*_2_, −0.23 [95% CI, −0.24 to −0.22]), reaching a change point at age 6 years (95% CI, 5.94-6.11), followed by linear increases from ages 6 to 9 years (phase 2: slope difference between phases [*b*_4_ − *b*_2_], 0.81 [95% CI, 0.76-0.86]). At age 9 years, the mean BMI was 17.33, which is approximately the 71st percentile for boys and the 67th percentile for girls. The 1006 children in the atypical group (10.6% of the sample) had a mean BMI of 17.87 (*b*_5_, 95% CI, 17.70-18.03) at age 1 year, which is roughly the 80th percentile for boys and the 85th percentile for girls. These children, on average, showed estimated stable BMIs from ages 1 to 3.5 years (*b*_6_, 0.06 [95% CI, −0.04 to 0.15]), followed by rapid linear increases from ages 3.5 to 9 years (slope difference [*b*_8_ − *b*_6_], 1.44 [95% CI, 1.34-1.55]). At age 9 years, the mean BMI was 26.2, which exceeded the 99th percentile for both boys and girls. The estimated age at the change point was 3.5 years (95% CI, 3.40-3.68).

**Figure.**
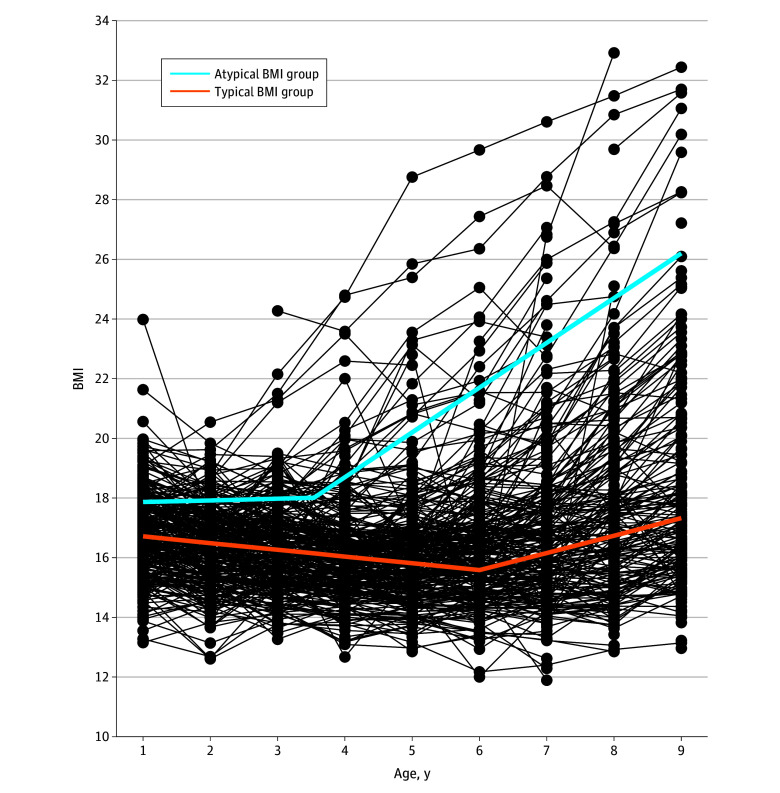
Body Mass Index (BMI) Trajectories From Ages 1 to 9 Years Among US Children in the Environmental influences on Child Health Outcomes Cohort Based on a Multiphase Latent Growth Mixture Model BMI was calculated as weight in kilograms divided by length in meters squared for children aged 1 and 2 years and as weight in kilograms divided by height in meters squared for children older than 2 years.

### Identification of Early-Life Factors Associated With BMI Trajectories

#### Class Membership

Compared with White children, Black children (OR, 2.42 [95% CI, 1.97-2.98]) and Hispanic or Latino children (OR, 2.48 [95% CI, 2.04-3.03) were more likely to be in the atypical group than the typical group, according to the covariates-only model. Girls (OR, 1.22 [95% CI, 1.05-1.42]) were more likely than boys to be in the atypical group. Prenatal smoking (OR, 1.76 [95% CI, 1.31-2.37]), higher vs lower prepregnancy BMI (OR, 1.09 [95% CI, 1.07-1.12]), and higher vs lower maternal gestational weight gain (OR, 1.26 [95% CI, 1.11-1.43]) were associated with a higher probability of being in the atypical group than the typical group. Children with higher vs lower birth weight (OR, 1.40 [95% CI, 1.21-1.63]) and preterm children (OR, 1.67 [95% CI, 1.24-2.25]) were more likely to be in the atypical group than the typical group. Children born to women with higher educational levels (OR, 0.85 [95% CI, 0.78-0.92]), prenatal alcohol exposure (OR, 0.79 [95% CI, 0.62-0.99), or children who were breastfed (OR, 0.75 [95% CI, 0.59-0.96]) were less likely to be in the atypical group. After including all exposure variables and covariates in the model, the associations with prenatal alcohol exposure, preterm status, and breastfeeding became nonsignificant. Detailed results are shown in Table 2, Table 3, and Table 4.

**Table 2.** Estimated Associations of Prenatal Exposures and Covariates With BMI Trajectories^a^

	Prenatal exposures–only model						Full model^b^ (including all exposures and covariates)					
	Class membership (C)	Change point, typical group; *b*_k1_	Change point, atypical group; *b*_k2_	Intercept in phase 1; *b*_1_/*b*_5_	Linear slope in phase 1; *b*_2_/*b*_6_	Slope differences between phase 2 and phase 1; (*b*_4_ − *b*_2_)/(*b*_8_ – *b*_6_)	Class membership (C)	Change point, typical group; *b*_k1_	Change point, atypical group; *b*_k2_	Intercept in phase 1; *b*_1_/*b*_5_	Linear slope in phase 1; *b*_2_/*b*_6_	Slope differences between phase 2 and phase 1; (*b*_4_ − *b*_2_)/(*b*_8_ – *b*_6_)
Child sex (boy vs girl)	1.22 (1.05 to 1.42)	0.03 (0.01 to 0.06)	−0.24 (−0.44 to −0.03)	−0.36 (−0.41 to −0.31)	0.04 (0.04 to 0.05)	−0.01 (−0.03 to 0.01)	1.27 (1.08 to 1.50)	0.02 (−0.01 to 0.05)	−0.24 (−0.44 to −0.04)	−0.31 (−0.35 to −0.26)	0.04 (0.03 to 0.05)	−0.01 (−0.03 to 0.02)
Race and ethnicity												
Black (compared with White)	2.28 (1.83 to 2.85)	0.07 (0.02 to 0.12)	0.15 (−0.14 to 0.44)	0.01 (−0.10 to 0.12)	0.02 (0.01 to 0.04)	0.13 (0.09 to 0.17)	1.87 (1.44 to 2.42)	0.05 (0.01 to 0.11)	0.22 (−0.07 to 0.52)	0.02 (−0.07 to 0.11)	0.02 (0.01 to 0.03)	0.11 (0.07 to 0.15)
Hispanic or Latino (compared with White)	2.63 (2.14 to 3.24)	−0.05 (−0.09 to −0.01)	−0.18 (−0.46 to 0.11)	0.02 (−0.05 to 0.10)	0.03 (0.02 to 0.04)	0.01 (−0.02 to 0.04)	2.47 (1.98 to 3.08)	−0.04 (−0.08 to −0.01)	−0.19 (−0.47 to 0.09)	0.04 (−0.03 to 0.11)	0.02 (0.01 to 0.03)	0.01 (−0.02 to 0.03)
Other (compared with White)^c^	1.18 (0.90 to 1.56)	0.05 (0.01 to 0.10)	0.20 (−0.22 to 0.62)	−0.26 (−0.34 to −0.18)	0.02 (0.01 to 0.03)	0.02 (−0.02 to 0.05)	1.25 (0.94 to 1.66)	0.04 (−0.01 to 0.08)	0.22 (−0.20 to 0.64)	−0.19 (−0.26 to −0.11)	0.02 (0.01 to 0.03)	0.02 (−0.01 to 0.06)
Maternal educational level during pregnancy	0.87 (0.80 to 0.95)	−0.01 (−0.02 to 0.01)	−0.01 (−0.10 to 0.11)	−0.06 (−0.09 to −0.03)	0.01 (0.01 to 0.01)	−0.01 (−0.02 to 0)	0.89 (0.81 to 0.99)	−0.01 (−0.02 to 0.02)	−0.01 (−0.11 to 0.11)	−0.06 (−0.09 to −0.04)	0.01 (0.01 to 0.01)	−0.01 (−0.02 to 0.01)
Prenatal exposure												
Smoking (no vs yes)	1.76 (1.31 to 2.37)	−0.03 (−0.09 to 0.03)	0.12 (−0.17 to 0.40)	0.23 (0.10 to 0.36)	−0.01 (−0.03 to −0.01)	0.03 (−0.02 to 0.09)	1.78 (1.26 to 2.52)	−0.05 (−0.11 to 0.02)	0.15 (−0.14 to 0.44)	0.26 (0.11 to 0.41)	−0.02 (−0.03 to −0.01)	0.03 (−0.02 to 0.09)
Alcohol use (no vs yes)	0.79 (0.62 to 0.99)	0.04 (−0.02 to 0.09)	0.02 (−0.27 to 0.31)	0.07 (−0.01 to 0.14)	−0.01 (−0.02 to −0.01)	0.02 (−0.01 to 0.05)	0.81 (0.64 to 1.02)	0.04 (−0.01 to 0.10)	−0.01 (−0.30 to 0.27)	0.03 (−0.04 to 0.10)	−0.01 (−0.02 to −0.01)	0.02 (−0.02 to 0.05)
Depression diagnosis (no vs yes)^d^	1.12 (0.85 to 1.48)	−0.01 (−0.07 to 0.05)	0.01 (−0.40 to 0.42)	0.06 (−0.19 to 0.30)	−0.01 (−0.03 to 0.03)	0.02 (−0.04 to 0.08)	0.97 (0.70 to 1.34)	0.01 (−0.06 to 0.07)	0.03 (−0.38 to 0.44)	0.01 (−0.18 to 0.18)	−0.01 (−0.03 to 0.03)	0.01 (−0.05 to 0.06)
Anxiety diagnosis (no vs yes)^d^	0.99 (0.65 to 1.50)	0.01 (−0.12 to 0.14)	−0.03 (−0.79 to 0.73)	0.09 (−0.15 to 0.33)	−0.01 (−0.04 to 0.02)	0.01 (−0.08 to 0.08)	0.98 (0.65 to 1.48)	0.01 (−0.12 to 0.15)	−0.02 (−0.79 to 0.76)	0.13 (−0.02 to 0.28)	−0.01 (−0.04 to 0.01)	0.01 (−0.07 to 0.07)

Abbreviation: BMI, body mass index (calculated as weight in kilograms divided by length in meters squared for children aged 1 and 2 years and as weight in kilograms divided by height in meters squared for children older than 2 years).

^a^ Data are presented as β (95% CI) except for class membership data, which are presented as odds ratio (95% CI). All coefficients are unstandardized; therefore, effects on change points are interpreted in years, and associations with BMI measures are interpreted in BMI units.

^b^ Includes all exposures and covariates.

^c^ Includes American Indian or Alaska Native, Asian, Native Hawaiian or Other Pacific Islander, and more than 1 race.

^d^ Includes perinatal exposure.

**Table 3.** Estimated Associations of Maternal Characteristics and Covariates With BMI Trajectories^a^

	Maternal characteristics–only model						Full model^b^ (including all exposures and covariates)					
	Class membership (C)	Change point, typical group; *b*_k1_	Change point, atypical group; *b*_k2_	Intercept in phase 1; *b*_1_/*b*_5_	Linear slope in phase 1; *b*_2_/*b*_6_	Slope differences between phase 2 and phase 1; (*b*_4_ − *b*_2_)/(*b*_8_ – *b*_6_)	Class membership (C)	Change point, typical group; *b*_k1_	Change point, atypical group; *b*_k2_	Intercept in phase 1; *b*_1_/*b*_5_	Linear slope in phase 1; *b*_2_/*b*_6_	Slope differences between phase 2 and phase 1; (*b*_4_ − *b*_2_)/(*b*_8_ – *b*_6_)
Child sex (boy vs girl)	1.23 (1.05 to 1.44)	0.03 (−0.01 to 0.06)	−0.23 (−0.43 to −0.02)	−0.35 (−0.40 to −0.31)	0.04 (0.04 to 0.05)	−0.01 (−0.03 to 0.01)	1.27 (1.08 to 1.50)	0.02 (−0.01 to 0.05)	−0.24(−0.44 to 0.04)	−0.31 (−0.35 to −0.26)	0.04 (0.03 to 0.05)	−0.01 (−0.03 to 0.02)
Race and ethnicity												
Black (compared with White)	1.92 (1.50 to 2.45)	0.09 (0.04 to 0.14)	0.20 (−0.08 to 0.48)	0.01 (−0.06 to 0.09)	0.01 (0.01 to 0.02)	0.12 (0.08 to 0.16)	1.87 (1.44 to 2.42)	0.05 (0.01 to 0.11)	0.22 (−0.07 to 0.52)	0.02 (−0.07 to 0.11)	0.02 (0.01 to 0.03)	0.11 (0.07 to 0.15)
Hispanic or Latino (compared with White)	2.29 (1.84 to 2.83)	−0.05 (−0.09 to −0.01)	−0.25 (−0.51 to 0.01)	−0.03 (−0.10 to 0.03)	0.03 (0.02 to 0.04)	0.01 (−0.03 to 0.02)	2.47 (1.98 to 3.08)	−0.04 (−0.08 to −0.01)	−0.19 (−0.47 to 0.09)	0.04 (−0.03 to 0.11)	0.02 (0.01 to 0.03)	0.01 (−0.02 to 0.03)
Other (compared with White)^c^	1.22 (0.92 to 1.61)	0.03 (−0.01 to 0.07)	0.16 (−0.26 to 0.59)	−0.27 (−0.34 to −0.20)	0.03 (0.02 to 0.04)	0.01 (−0.02 to 0.04)	1.25 (0.94 to 1.66)	0.04 (−0.01 to 0.08)	0.22 (−0.20 to 0.64)	−0.19 (−0.26 to −0.11)	0.02 (0.01 to 0.03)	0.02 (−0.01 to 0.06)
Maternal educational level during pregnancy	0.88 (0.80 to 0.96)	−0.01 (−0.02 to 0.01)	−0.01 (−0.11 to 0.11)	−0.06 (−0.09 to −0.04)	0.01 (0.01 to 0.01)	−0.01 (−0.02 to 0)	0.89 (0.81 to 0.99)	−0.01 (−0.02 to 0.02)	−0.01 (−0.11 to 0.11)	−0.06 (−0.09 to −0.04)	0.01 (0.01 to 0.01)	−0.01 (−0.02 to 0.01)
Maternal characteristic												
Prepregnancy BMI	1.09 (1.07 to 1.12)	−0.01 (−0.01 to −0.01)	−0.01 (−0.03 to 0.01)	0.02 (0.01 to 0.02)	0.01 (0.01 to 0.01)	0.01 (0.01 to 0.01)	1.09 (1.06 to 1.12)	−0.01 (−0.01 to −0.01)	−0.01 (−0.03 to 0.01)	0.02 (0.01 to 0.02)	0.01 (0.01 to 0.01)	0.01 (0.01 to 0.01)
Gestational weight gain	1.26 (1.11 to 1.43)	−0.04 (−0.06 to −0.01)	−0.03 (−0.18 to 0.13)	0.16 (0.12 to 0.19)	0 (−0.01 to 0.01)	0.02 (0.01 to 0.04)	1.21 (1.06 to 1.37)	−0.02 (−0.04 to 0.01)	−0.03 (−0.19 to 0.13)	0.07 (0.03 to 0.11)	0.01 (−0.01 to 0.01)	0.01 (−0.01 to 0.03)

Abbreviation: BMI, body mass index (calculated as weight in kilograms divided by length in meters squared for children aged 1 and 2 years and as weight in kilograms divided by height in meters squared for children older than 2 years).

^a^ Data are presented as β (95% CI) except for class membership data, which are presented as odds ratio (95% CI). All coefficients are unstandardized; therefore, effects on change points are interpreted in years, and associations with BMI measures are interpreted in BMI units.

^b^ Includes all exposures and covariates.

^c^ Includes American Indian or Alaska Native, Asian, Native Hawaiian or Other Pacific Islander, and more than 1 race.

**Table 4.** Estimated Associations of Child Characteristics and Covariates With BMI Trajectories^a^

	Child characteristics–only model						Full model^b^ (including all exposures and covariates)					
	Class membership (C)	Change point, typical group; *b*_k1_	Change point, atypical group; *b*_k2_	Intercept in phase 1; *b*_1_/*b*_5_	Linear slope in phase 1; *b*_2_/*b*_6_	Slope differences between phase 2 and phase 1; (*b*_4_ − *b*_2_)/(*b*_8_ – *b*_6_)	Class membership (C)	Change point, typical group; *b*_k1_	Change point, atypical group; *b*_k2_	Intercept in phase 1; *b*_1_/*b*_5_	Linear slope in phase 1; *b*_2_/*b*_6_	Slope differences between phase 2 and phase 1; (*b*_4_ − *b*_2_)/(*b*_8_ – *b*_6_)
Child sex (boy vs girl)	1.27 (1.09 to 1.48)	0.02 (−0.01 to 0.05)	−0.24 (−0.44 to −0.03)	−0.31 (−0.35 to −0.26)	0.04 (0.04 to 0.05)	−0.01 (−0.03 to 0.02)	1.27 (1.08 to 1.50)	0.02 (−0.01 to 0.05)	−0.24 (−0.44 to −0.04)	−0.31 (−0.35 to −0.26)	0.04 (0.03 to 0.05)	−0.01 (−0.03 to 0.02)
Race and ethnicity												
Black (compared with White)	2.37 (1.88 to 2.99)	0.04 (0.01 to 0.10)	0.21 (−0.08 to 0.50)	0.07 (−0.01 to 0.15)	0.02 (0.01 to 0.03)	0.13 (0.09 to 0.17)	1.87 (1.44 to 2.42)	0.05 (0.01 to 0.11)	0.22 (−0.07 to 0.52)	0.02 (−0.07 to 0.11)	0.02 (0.01 to 0.03)	0.11 (0.07 to 0.15)
Hispanic or Latino (compared with White)	2.57 (2.10 to 3.14)	−0.06 (−0.10 to −0.03)	−0.27 (−0.53 to −0.02)	0.01 (−0.05 to 0.08)	0.03 (0.02 to 0.04)	0.01 (−0.02 to 0.03)	2.47 (1.98 to 3.08)	−0.04 (−0.08 to −0.01)	−0.19 (−0.47 to 0.09)	0.04 (−0.03 to 0.11)	0.02 (0.01 to 0.03)	0.01 (−0.02 to 0.03)
Other (compared with White)^c^	1.23 (0.94 to 1.61)	0.02 (−0.02 to 0.07)	0.17 (−0.24 to 0.59)	−0.23 (−0.30 to −0.16)	0.03 (0.02 to 0.04)	0.02 (−0.02 to 0.05)	1.25 (0.94 to 1.66)	0.04 (−0.01 to 0.08)	0.22 (−0.20 to 0.64)	−0.19 (−0.26 to −0.11)	0.02 (0.01 to 0.03)	0.02 (−0.01 to 0.06)
Maternal educational level during pregnancy	0.85 (0.78 to 0.92)	−0.01 (−0.01 to 0.02)	−0.01 (−0.11 to 0.10)	−0.08 (−0.11 to −0.05)	0.01 (0.01 to 0.01)	−0.01 (−0.02 to −0.01)	0.89 (0.81 to 0.99)	−0.01 (−0.02 to 0.02)	−0.01 (−0.11 to 0.11)	−0.06 (−0.09 to −0.04)	0.01 (0.01 to 0.01)	−0.01 (−0.02 to 0.01)
Child characteristic												
Breastfeeding (no vs yes)	0.75 (0.59 to 0.96)	−0.03 (−0.10 to 0.03)	0.18 (−0.14 to 0.49)	−0.27 (−0.37 to −0.18)	0.02 (0.01 to 0.03)	−0.05 (−0.09 to −0.01)	0.87 (0.67 to 1.15)	−0.04 (−0.10 to 0.03)	0.26 (−0.07 to 0.58)	−0.19 (−0.30 to −0.09)	0.02 (−0.01 to 0.03)	−0.03 (−0.08 to 0.02)
Preterm status (no vs yes)	1.67 (1.24 to 2.25)	−0.06 (−0.13 to 0.02)	−0.44 (−0.88 to 0.03)	0.35 (0.21 to 0.50)	−0.01 (−0.03 to 0.01)	0.02 (−0.04 to 0.07)	1.30 (0.92 to 1.84)	−0.04 (−0.11 to 0.04)	−0.35 (−0.80 to 0.09)	0.32 (0.19 to 0.46)	−0.02 (−0.04 to −0.01)	−0.01 (−0.05 to 0 .05)
Birth weight, kg	1.40 (1.21 to 1.63)	−0.09 (−0.12 to −0.05)	−0.11 (−0.31 to 0.08)	0.45 (0.38 to 0.53)	−0.02 (−0.02 to −0.01)	0.04 (0.02 to 0.07)	1.28 (1.09 to 1.49)	−0.08 (−0.12 to −0.05)	−0.08 (−0.28 to 0.11)	0.43 (0.35 to 0.51)	−0.02 (−0.03 to −0.01)	0.03 (0.01 to 0.06)

Abbreviation: BMI, body mass index (calculated as weight in kilograms divided by length in meters squared for children aged 1 and 2 years and as weight in kilograms divided by height in meters squared for children older than 2 years).

^a^ Data are presented as β (95% CI) except for class membership data, which are presented as odds ratio (95% CI). All coefficients are unstandardized; therefore, effects on change points are interpreted in years, and associations with BMI measures are interpreted in BMI units.

^b^ Includes all exposures and covariates.

^c^ Includes American Indian or Alaska Native, Asian, Native Hawaiian or Other Pacific Islander, and more than 1 race.

#### Change Point

We allowed the estimation of the change point age to vary across groups because previous analyses had already indicated significant group differences in its timing. Of note, individual differences in the change point were mainly explained by group membership (typical vs atypical). After controlling for these between-group effects, we examined within-group variance factors. For the typical group, Hispanic or Latino children showed an earlier change point than White children (β, −0.06 [95% CI, −0.09 to −0.02]), according to the covariates-only model, representing a difference of approximately 22 days, whereas Black children showed a later change point than White children (β, 0.07 [95% CI, 0.02-0.12]), representing a difference of 26 days. While statistically significant, this timing difference is small, and its clinical significance should be interpreted cautiously. However, these small differences can have meaningful population-level outcomes when considering their cumulative effects across large groups of children and their potential influence on long-term health trajectories. Girls showed a later change point than boys (β, 0.03 [95% CI, 0.01-0.06]). Higher prepregnancy BMI (β, −0.01 [95% CI, −0.01 to −0.01]) and higher gestational weight gain (β, −0.04 [95% CI, −0.06 to −0.01]) were associated with an earlier change point. Children with higher birth weights showed an earlier change point (β, −0.09 [95% CI, −0.12 to −0.05]). For the atypical group, girls had an earlier adiposity rebound than boys (β, −0.25 [95% CI, −0.43 to −0.03]). After including all exposure variables and covariates in the model, for the typical group, gestational weight gain and child sex became nonsignificant factors.

#### Growth Parameters

The associations of the early-life factors on the growth parameters were constrained to be the same across groups. Prenatal smoking (β, 0.23 [95% CI, 0.10-0.36]), higher prepregnancy BMI (β, 0.02 [95% CI, 0.01-0.02]), higher gestational weight gain (β, 0.16 [95% CI, 0.12-0.19]), preterm status (β, 0.35 [95% CI, 0.21-0.50]), and higher birth weight (β, 0.45 [95% CI, 0.38-0.53]) were associated with higher initial levels of BMI in phase 1. Girl (vs boy) (β, −0.36 [95% CI, −0.41 to −0.31]), higher maternal educational level (β, −0.07 [95% CI, −0.10 to −0.04]), and breastfeeding (β, −0.27 [95% CI, −0.37 to −0.18]) were associated with lower initial BMI levels in phase 1. For the linear slope in phase 1, Black children compared with White children (β, 0.02 [95% CI, 0.01-0.03]), Hispanic or Latino children compared with White children (β, 0.03 [95% CI, 0.02-0.04]), girl vs boy (β, 0.04 [95% CI, 0.04-0.05]), higher maternal educational level (β, 0.01 [95% CI, 0.01-0.01]), higher prepregnancy BMI (β, 0.01 [95% CI, 0.01-0.01), and breastfeeding (β, 0.02 [95% CI, 0.01-0.03]) were associated with larger linear slopes (ie, slower decreases or faster increases). Prenatal smoking (β, −0.01 [95% CI, −0.03 to −0.01]), prenatal alcohol use (β, −0.01 [95% CI, −0.02 to −0.01]), and higher vs lower birth weight (β, −0.02 [95% CI, −0.02 to −0.01]) were associated with smaller linear slopes in phase 1 (ie, faster decreases or slower increases). After including all variables in the model, the association between breastfeeding and slope in phase 1 became nonsignificant.

For the slope differences between phases 2 and 1, Black children compared with White children (β, 0.14 [95% CI, 0.10-0.17]), higher prepregnancy BMI (β, 0.01 [95% CI, 0.01-0.01]), higher gestational weight gain (β, 0.02 [95% CI, 0.01-0.04]), and higher birth weight (β, 0.04 [95% CI, 0.02-0.07]) were associated with more rapid BMI increases in phase 2 compared with phase 1. Higher maternal educational level (β, −0.01 [95% CI, −0.02 to −0.01]) and breastfeeding (β, −0.05 [95% CI, −0.09 to −0.01]) were associated with a less rapid BMI increase in phase 2 compared with phase 1. After including all variables in the model, maternal educational level, gestational weight gain, and breastfeeding became nonsignificant.

### Subsequent Analysis

To investigate potential distinct BMI trajectories between boys and girls from ages 1 to 9 years, we conducted separate analyses by child sex. For the single-phase model, the results replicated the findings based on the full group, in which a 2-class model was selected as the optimal model for both girls and boys. Regarding the multiphase model, the results were consistent with the findings based on the full group (eResults in Supplement 1).

## Discussion

To our knowledge, this study is one of the first to systematically characterize multiphase BMI trajectories from toddlerhood to preadolescence and assess individual differences (eg, atypicality) in within-person transitions through developmental phases. We identified 2 distinct 2-phase BMI trajectories: a typical pattern (89.4%) with BMI decreases from ages 1 to 6 years followed by gradual increases and an atypical pattern (10.6%) with stable BMI from ages 1 to 3.5 years followed by rapid increases. Most children in the atypical group developed obesity by age 9 years, with a group mean BMI above the 99th percentile. The findings on the typical trajectory are consistent with previous work using different approaches^47,48^ or a similar multiphase^49^ model. Importantly, we identified children who deviated from the typical trajectory and showed a no-decline trajectory with an early BMI increase, potentially facing the highest obesity risks.^19,20,50,51,52^ The current study addressed the limitations of previous work that focused on typical development (ie, normative decline and adiposity rebound patterns) and that excluded children with atypical trajectories from further analysis.^17,52^ For example, Wen et al^17^ were unable to identify the adiposity rebound for a subgroup of children who bypassed the normative decline phase and did not show a typical decrease-increase trajectory. The findings of the present study could facilitate early identification of children who are most likely to develop obesity and inform optimal timing for interventions.

The multiphase growth mixture model is well suited for research where timing of individual-specific change points and phase transitions are key interests. Compared with the traditionally used visual inspection of change point method,^53^ where researchers visually pinpoint the nadir of the BMI trajectory directly from observed data, the multiphase approach offers several advantages.^49^ The visual inspection method can only estimate change points at ages where BMI measurements are available, and its accuracy depends on the number of BMI assessments and intervals between them, limiting the ability to freely estimate the nadir or change point.^53^ In contrast, the multiphase growth mixture model enables the estimated nadir or change point to occur between observed BMI assessment time points, providing greater precision in identifying phase transitions. Another advantage of the current model is that estimated parameters, such as the individual-specific change points and rates of change, are easily interpretable. However, interpreting change point and slope estimates in typically-used models, such as mixed-effects models with polynomial functions, is often problematic.^49^ Moreover, the generalizability of these typically-used models becomes questionable once they are increasingly tailored to specific data patterns (a detailed discussion is provided in the eDiscussion in Supplement 1).

In this study, we examined whether early-life factors were associated with between-group (typical vs atypical) and within-group differences. Consistent with previous work,^54,55^ we found that Black and Hispanic or Latino children, compared with White children, were more likely to be in the atypical group with an early BMI increase, indicating potential racial and ethnic disparities in childhood obesity risk. Black children also showed more rapid BMI increases during the rebound phase. Hispanic or Latino children in the typical group exhibited an earlier BMI increase during the rebound phase compared with White children. This suggests that Black and Hispanic or Latino children with healthy BMI trajectories may still struggle and be at increased risk of later obesity, necessitating careful monitoring over time. The observed racial and ethnic disparities may be attributed to differences in social and environmental factors, including gaps in family and neighborhood socioeconomic status, lack of access to healthy food, and chronic stress associated with discrimination. These factors may disproportionately expose racially and ethnically minoritized children to obesity risk factors from an early age.^33,38,39,56,57^

Our study identified several key risk factors for childhood obesity: prenatal smoking, high prepregnancy BMI, high gestational weight gain, and high birth weight. Our finding that prenatal smoking was a key risk factor for child obesity has been consistently identified in previous work,^51,58^ although confounding by obesity risk factors associated with maternal smoking remains a concern. In this study, we controlled for many potential confounders and identified the independent association of prenatal smoking with child BMI trajectories. In line with previous work,^51,59^ high maternal prepregnancy BMI was a key factor across all parameters, including earlier adiposity rebound, setting the stage for persistent obesity and related health issues for children. When examining maternal factors alone, gestational weight gain was associated with all estimated parameters. However, some of these associations became nonsignificant after including other exposures, indicating that its associations with childhood obesity risks may be explained by related factors such as birth weight and prenatal smoking.^49,60^ High birth weight consistently was associated with an increased risk of childhood obesity, supporting the hypothesis that fetal overgrowth and overnutrition can have long-lasting associations with children’s growth, placing children on an accelerated growth trajectory.^61^

We also identified potential protective factors against childhood obesity. Higher maternal educational level was associated with decreased obesity risk, emphasizing the importance of social determinants of health. Regarding breastfeeding, the initial analyses indicated a potentially protective role, with lower likelihood of childhood obesity. However, these associations became nonsignificant after including other factors, which is consistent with previous research.^62,63^

This study used data from the ECHO cohort, which provided several unique advantages. First, ECHO included children from diverse racial and ethnic, geographical, and socioeconomic backgrounds from 69 cohorts across 44 states and Puerto Rico, providing greater generalizability than previous single-site or regionally focused studies. The large, demographically diverse sample allowed us to examine BMI trajectories across different racial and ethnic groups, addressing an important gap in the literature, as most previous studies have focused on predominantly White participants. Additionally, the breadth of prenatal and early-life data collected across the ECHO cohort enabled comprehensive examination of multiple risk and protective factors that may have influenced BMI trajectories.

### Clinical Implications and Future Directions

Our findings may have important implications for clinical practice and public health interventions. The identification of atypical BMI trajectories as early as age 3.5 years suggests that early childhood may represent a critical window for obesity prevention. The findings suggest potential opportunities to decrease childhood obesity. One opportunity is to target individuals of reproductive age with strategies aimed at smoking cessation and achievement of a healthy body weight before conception. An additional opportunity is to target pregnant individuals to reduce prenatal smoking and achieve healthy weight gain during pregnancy. A third opportunity is to closely monitor children showing early signs of accelerated BMI increase, particularly those exposed to identified risk factors such as prenatal smoking, high maternal prepregnancy BMI, and excessive gestational weight gain. Families with children showing nondeclining trajectories or very early BMI increase may benefit from intensive prevention and intervention programs focusing on healthy food choices and physical activity. Future research is needed to examine potential biological mechanisms linking early-life factors to child BMI trajectories and investigate how social and environmental factors explain racial and ethnic disparities in childhood obesity risk.

### Limitations

The findings from this study should be interpreted considering the following limitations. First, the clinical utility of the multiphase models remains unclear. These methods are utilized in research settings and tend to be more computationally intensive than traditional methods of identifying abnormal childhood growth patterns (ie, crossing of major percentile lines on a standard growth chart). Second, the BMI values used in this study may represent different levels and distributions of adiposity depending on a child’s age and sex. In this study, we provided BMI percentile values alongside the raw BMI values to aid in the interpretation of the findings, as BMI percentiles account for age and sex differences and provide more clinically relevant information. Third, although this study controlled for some social determinants of health (child race and ethnicity and maternal educational level), it did not include other important factors such as household income, food insecurity, neighborhood characteristics, and access to health care,^64,65,66^ which may influence child BMI trajectories. Moreover, several other early-life factors associated with childhood obesity in previous research were not examined in our study. These include maternal diet during pregnancy, maternal physical activity during pregnancy, infant sleep patterns, infant feeding behaviors, and timing of complementary feeding. Future research is needed to conduct a more comprehensive examination of early-life factors and social determinants of child BMI trajectories. Fourth, this study has large missing data on BMI assessments during the school-age period (eg, 73.0% missingness for age 9-year assessment) and exposure variables (eg, 41.2% missingness for prenatal or perinatal depression diagnosis). For the multiphase latent growth mixture model (to identify BMI trajectories), we used the FIML estimation to handle missing BMI assessments, which is appropriate for longitudinal analyses when data are missing at random. We required children to have at least 4 BMI assessments for inclusion, and our sample included 53 152 total BMI observations. The FIML estimation has been shown to be effective for longitudinal data with high rates of missing data when there are large numbers of observations (eg, N = 20 000).^67^ For the exposure variables and covariates, we used multiple imputation to handle missing data, which is appropriate for missing rates around 50%.^68^ Nonetheless, we acknowledge that the high percentage of missing data may have affected the precision of our estimates, which is a possible inadequacy of the FIML estimation in handling nonignorable missingness due to omission of other key variables.^69,70,71^ Fifth, while this study includes participants from the ECHO cohort, including children from diverse racial and ethnic, geographical, and socioeconomic backgrounds, the findings may not be fully generalizable to the US population, as participation in longitudinal studies often requires resources and commitment that may limit participation from the most resource-limited families. Sixth, although 95.8% of child BMI measurements came from medical records and staff measurements, a small portion (4.2%) relied on caregiver reports, remote study measures, or self-report, which may have introduced some measurement error. Seventh, breastfeeding was assessed as ever breastfed or not, which limited assessment of this exposure, since many individuals who begin breastfeeding stop within the first few weeks. A more comprehensive measure of breastfeeding, including the duration, exclusivity, and intensity of breastfeeding, would be preferable for attempting to replicate this study’s findings.

## Conclusions

In this cohort study that used a novel modeling approach, we identified 2 distinct 2-phase BMI patterns that distinguished children on an early path to obesity from those with normative development as early as age 3.5 years. We also identified modifiable early-life factors that may place children at risk for or protect children from childhood obesity. Intervention programs could focus on modifiable factors (eg, prenatal smoking cessation, healthy prepregnancy weight, appropriate gestational weight gain) and target children showing atypical BMI trajectories for intensive prevention and intervention efforts. Addressing these factors may help redirect unhealthy BMI trajectories, potentially playing a crucial role in childhood obesity prevention.

## Notes

Supplement 1. **eTable 1.** Participant Demographics **eTable 2.** Assessment Methods and Data Sources for Exposures **eMethods 1.** Model Specification **eMethods 2.** Multiple Imputation Specification **eTable 3.** Single-Phase Latent Growth Mixture Model Overall Model Fit Statistics **eFigure.** BMI Trajectories From Ages 1 to 9 Years Among US Children in the ECHO Cohort Based on a Two-Class Single-Phase Latent Growth Mixture Model **eResults.** Separate Analysis Results for Boys and Girls **eDiscussion.** Limitations of Previous Models and Advantages of the Multiphase Latent Growth Mixture Model

Supplement 2.
Nonauthor Collaborators

Supplement 3.
Data Sharing Statement
