# Supplementary material for: Early-Life Factors and Body Mass Index Trajectories Among Children in the ECHO Cohort
Source: JAMA Netw Open. 2025 May 22;8(5):e2511835. doi: 10.1001/jamanetworkopen.2025.11835 (PMC12100454; doi:10.1001/jamanetworkopen.2025.11835)
Supplement: Supplement 1. — eTable 1. Participant Demographics eTable 2. Assessment Methods and Data Sources for Exposures eMethods 1. Model Specification eMethods 2. Multiple Imputation Specification eTable 3. Single-Phase Latent Growth Mixture Model Overall Model Fit Statistics eFigure. BMI Trajectories From Ages 1 to 9 Years Among US Children in the ECHO Cohort Based on a Two-Class Single-Phase Latent Growth Mixture Model eResults. Separate Analysis Results for Boys and Girls eDiscussion. Limitations of Previous Models and Advantages of the Multiphase Latent Growth Mixture Model [file jamanetwopen-e2511835-s001.pdf]

Supplemental Online Content

Liu C, Chow SM, Aris IM, et al; on behalf of Environmental Influences on Child Health Outcomes (ECHO). Early-life factors and body mass index trajectories among children in the ECHO cohort. *JAMA Netw Open*. 2025;8(5):e2511835. doi:10.1001/jamanetworkopen.2025.11835

eTable 1. Participant Demographics
eTable 2. Assessment Methods and Data Sources for Exposures
eMethods 1. Model Specification
eMethods 2. Multiple Imputation Specification
eTable 3. Single-Phase Latent Growth Mixture Model Overall Model Fit Statistics
eFigure. BMI Trajectories From Ages 1 to 9 Years Among US Children in the ECHO Cohort Based on a Two-Class Single-Phase Latent Growth Mixture Model
eResults. Separate Analysis Results for Boys and Girls
eDiscussion. Limitations of Previous Models and Advantages of the Multiphase Latent Growth Mixture Model

This supplemental material has been provided by the authors to give readers additional information about their work.

© 2025 Liu C et al. *JAMA Network Open*.

eTable 1. Participant Demographics

	Full Sample (*N* = 9483): No. (Percentage)	Typical Group (*n* = 8477): No. Percentage	Atypical Group (*n* = 1006): No. Percentage
**Child Sex**			
boy	4925 (51.9)	4442 (52.4)	483 (48.0)
girl	4558 (48.1)	4035 (47.6)	523 (52.0)
**Child Race/Ethnicity**			
Hispanic/Latino	1854 (19.6)	1569 (18.5)	285 (28.3)
Non-Hispanic Black	1762 (18.6)	1487 (17.5)	275 (27.3)
Non-Hispanic White	4358 (46.0)	4044 (47.7)	314 (31.2)
Non-Hispanic Others[a]	1318 (13.8)	1212 (14.3)	106 (10.5)
Missing	191 (2.0)	165 (2.0)	26 (2.7)
**Maternal Educational Level During Pregnancy**			
Less than high school	264 (2.8)	213 (2.5)	51 (5.1)
High school degree, GED or equivalent	904 (9.5)	778 (9.2)	126 (12.5)
Some college	1289 (13.6)	1105 (13.0)	184 (18.3)
Bachelor's degree	1402 (14.8)	1295 (15.3)	107 (10.6)
Professional or Doctorate Degree	992 (10.5)	946 (11.2)	46 (4.6)
Missing	4632 (48.9)	4140 (48.8)	492 (48.9)
**Birth Years**			
1997–2000	276 (2.9)	260 (3.1)	16 (1.6)
2001–2005	1392 (14.7)	1211 (14.3)	181 (18.0)
2006–2010	2066 (21.8)	1853 (21.9)	213 (21.2)
2011–2015	4265 (45)	3798 (44.8)	467 (46.4)
2016–2019	1484 (15.7)	1355 (16.0)	129 (12.8)

[a]Non-Hispanic Others include American Indian or Alaska Native, Asian, Native Hawaiian or other Pacific Islander, and more than one race.

© 2025 Liu C et al. *JAMA Network Open*.

eTable 2. Assessment Methods and Data Sources for Exposures

	Frequency No. (%)	Missing (%)
**Prenatal Exposure**		
Smoking		996 (10.5)
Self-report	7418 (78.2)	
Medical records	4390 (46.3)	
Alcohol use		1034 (10.9)
Self-report	6930 (73.1)	
Medical records	3391 (35.8)	
Depression diagnosis		3908 (41.2)
Self-report	139 (1.5)	
Medical records	5436 (57.3)	
Anxiety diagnosis		4952 (52.2)
Self-report	0 (0)	
Medical records	4531 (47.8)	
**Maternal Characteristics**		
Pre-pregnancy weight (kg)		1510 (15.9)
Self-report	2895 (30.5)	
Medical records	2428 (25.6)	
Study measure	2650 (27.9)	
Height (cm)		1198 (12.6)
Self-report	1626 (17.2)	
Medical records	1553 (16.4)	
Study measure	5106 (53.8)	
Gestational weight gain		2396 (25.3)
Self-report	2444 (25.8)	
Medical records	4206 (44.4)	
Study measure	437 (4.6)	
**Child Characteristics**		
Breastfeeding		1545 (16.3)
Self-report	7938 (83.7)	
Child preterm status		113 (1.2)
Mother or caregiver report	2984 (31.5)	
Medical records	5684 (59.9)	
Study measure	437 (4.6)	
Other data source	265 (2.8)	
Birthweight (kg)		76 (0.8)
Mother report	1646 (17.4)	
Other report	1153 (12.2)	
Medical records	6584 (69.4)	
Study measure	24 (2.5)	

© 2025 Liu C et al. *JAMA Network Open*.

eMethods 1. Model Specification

The model is specified below. To allow for the statistical properties (e.g., algebraic form) and change point of the multiphase model to vary based on the individual's latent class, we used a latent categorical variable, C_j, to designate each individual j's class membership, which was assumed to be invariant over time. We allowed for person-specific random effects (i.e., individual differences) in the intercepts and slopes of the BMI growth and the change point:

For latent class 1 ($C_j = 1$), the developmental trajectories are partitioned into 2 phases, and there is 1 change point, denoted as b_{k1j}:

$$BMI_{ij} = \begin{cases} b_{1j} + b_{2j}t_{ij} + \varepsilon_{ij} & t_{ij} \leq b_{k1j} \\ b_{4j}t_{ij} + \varepsilon_{ij} & t_{ij} > b_{k1j} \end{cases} \tag{1}$$

For latent class 2 ($C_j = 2$), the developmental trajectories are partitioned into 2 phases, and there is 1 change point, denoted as b_{k2j}:

$$BMI_{ij} = \begin{cases} b_{5j} + b_{6j}t_{ij} + \varepsilon_{ij} & t_{ij} \leq b_{k2j} \\ b_{8j}t_{ij} + \varepsilon_{ij} & t_{ij} > b_{k2j} \end{cases} \tag{2}$$

where t_{ij} is the age i for individual j; b_{1j} and b_{2j} are the intercept and slope for the first phase for individual j in Class 1, respectively; b_{4j} is the slope for the second phase for individual j in Class 1; b_{5j} and b_{6j} are the intercept and slope for the first phase for individual j in Class 2, respectively; b_{8j} is the slope for the second phase for individual j in Class 2; b_{k1} is the change point for individual j in Class 1; b_{k2} is the change point for individual j in Class 2; and ε_{ij} is the residual error. We dropped out the intercepts for the second phase b_{3j} and b_{7j}, because the two phases joined at the change point, and the intercepts of the second phase can be calculated based on the other growth coefficient estimates.

© 2025 Liu C et al. *JAMA Network Open*.

We allowed the growth rates in each phase and the change point to vary across classes and individuals. However, we constrained class membership to be invariant across individuals. That is, individuals were allowed to transition across multiple phases within a class but not change class membership over time. In addition, some interindividual differences were assumed to be present in the intercepts and linear slopes across classes in Phase 1, any subsequent across-phase differences in slopes, and in the change points, with the means of all growth curve-related parameters allowed to differ between classes.

© 2025 Liu C et al. *JAMA Network Open*.

eMethods 2. Multiple Imputation Specification

Consistent with previous research, we used full information maximum likelihood estimation (FIML) to handle missing BMI assessments in the multiphase latent growth mixture model. We used multiple imputation with chained equations (MICE) to address missing data in exposure variables and covariates. MICE automatically selected appropriate imputation methods based on variable type: Predictive Mean Matching for continuous variables, Logistic Regression for binary variables, and Multinomial Logistic Regression for categorical variables with more than two levels. We used 20 imputations, and the imputed datasets were analyzed in *Mplus*, which automatically implements Rubin's rules to combine results across imputations. Specifically, the parameter estimates were averaged across imputed datasets, and the standard errors were calculated by combining both within- and between-imputation variance components following Rubin's method (Rubin, 1987). The confidence intervals were then constructed based on these combined estimates and standard errors. This approach provides pooled parameter estimates and appropriate standard errors that account for both within- and between-imputation uncertainty.

© 2025 Liu C et al. *JAMA Network Open*.

eTable 3. Single-Phase Latent Growth Mixture Model Overall Model Fit Statistics

	Classes		
	**1-class model**	**2-class model**	**3-class model**
AIC	185299	181731	180485
BIC	185464	181932	180721
saBIC	185391	181843	180616
Entropy		.96	.92
BLRT improvement		<.01	<.01
Number of children/class (% of total sample)			
1		587 (6.2)	243 (2.6)
2		8896 (93.8)	905 (9.5)
3			8335 (87.9)

Abbreviations: AIC, Akaike information criterion; BIC, Bayesian information criterion; BLRT, bootstrap likelihood ratio test; saBIC, size-adjusted Bayesian information criterion

© 2025 Liu C et al. *JAMA Network Open*.

eFigure. BMI Trajectories From Ages 1 to 9 Years Among US Children in the ECHO Cohort Based on a Two-Class Single-Phase Latent Growth Mixture Model

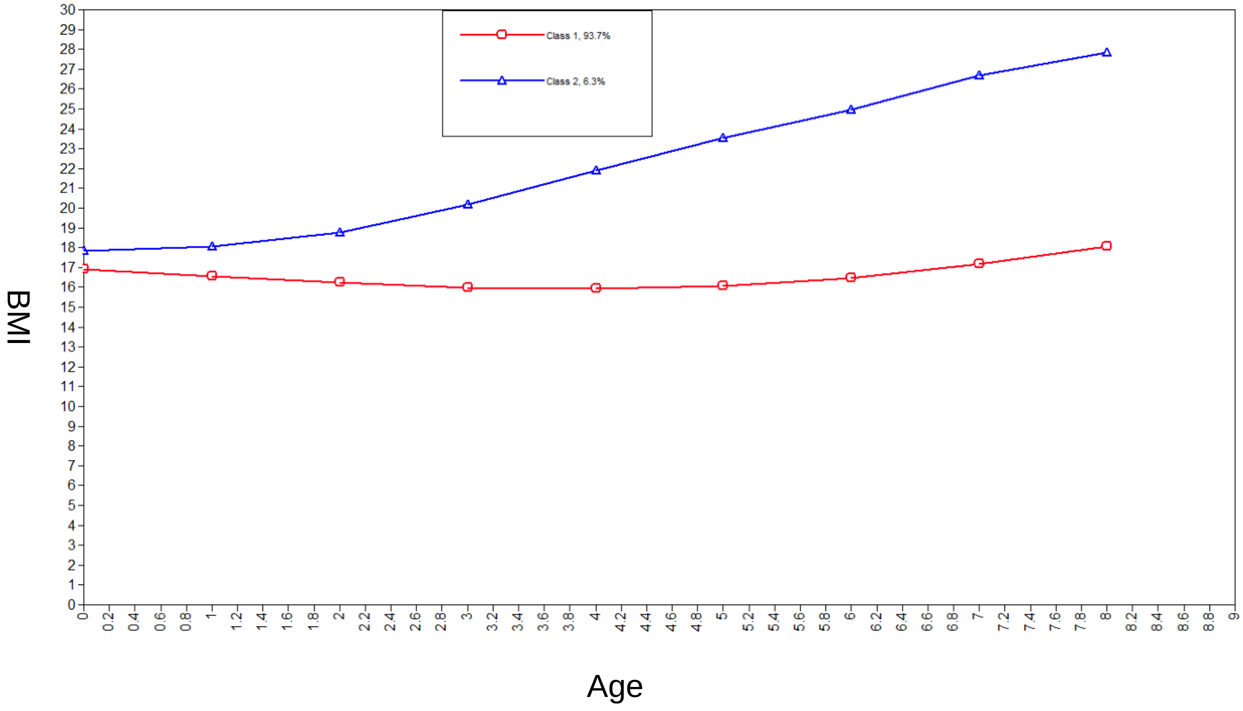

Note. On the x-axis, 0 represents age 1 and 8 represents age 9.

© 2025 Liu C et al. *JAMA Network Open*.

eResults. Separate Analysis Results for Boys and Girls

To investigate potential distinct BMI growth trajectories between girls and boys from 1 to 9 years, we conducted separate analyses for girls and boys, including both single-phase and multiphase latent growth mixture models. For the single-phase model, the results replicated the findings based on the full group, where a 2-class model was selected as the optimal model for both boys and girls. Regarding the multiphase model, the results were consistent with the findings based on the full group. Specifically, for girls, children in Group 1 (the typical group, 89.9% of the sample, n = 4097) had an average BMI of 16.44 kg/m^2 at age 1 (95% confidence interval (CI), 16.38 to 16.50). Subsequently, these children showed linear BMI decreases from ages 1 to 6.1 years (Phase 1; b_2 = -0.17; 95%CI, -0.19 to -0.16), reaching a change point at age 6.1 years (95%CI, 5.93 to 6.20), followed by linear increases from ages 6.1 to 9 years (Phase 2; slope difference between phases: b_4 - b_2 = 0.88; 95%CI, 0.79 to 0.96). At age 9, the average BMI was 17.63 kg/m^2 (95%CI, 17.42 to 17.84). Children in Group 2 (the atypical group, 10.1% of the sample, n = 461) had an average BMI of 18.1 kg/m^2 at age 1 (95%CI, 17.79 to 18.47). Subsequently, these children, on average, showed decreased BMI from ages 1 to 2.5 years (b_6 = -0.48; 95%CI, -0.79 to -0.17), reaching a change point at 2.5 years (95%CI, 2.28 to 2.67), followed by rapid linear increases from ages 2.5 to 9 years (slope difference b_8 - b_6 = 1.65; 95%CI, 1.38 to 1.93). At age 9, the average BMI was 25.11 kg/m^2 (95%CI, 24.07 to 26.15). Similarly, for boys, children in Group 1 (the typical group, 89.2% of the sample, n = 4395) had an average BMI of 16.96 kg/m^2 at age 1 (95%CI, 16.91 to 17.01). These children subsequently showed linear BMI decreases from ages 1 to 6.1 years (Phase 1; b_2 = -0.27; 95%CI, -0.28 to -0.26), reaching a change point at age 6.1 years (95%CI, 5.98 to 6.18), followed by linear increases from ages 6.1 to 9 years (Phase 2; slope difference between phases: b_4 - b_2 = 0.82;

© 2025 Liu C et al. *JAMA Network Open*.

95%CI, 0.76 to 0.89). At age 9, the average BMI was 17.22 kg/m^2 (95%CI, 17.05 to 17.39). Children in Group 2 (the atypical group, 10.8% of the sample, *n* = 530) had an average BMI of 17.98 kg/m^2 at age 1 (95%CI, 17.76 to 18.20). Subsequently, these children, on average, showed stable BMI from ages 1 to 3.6 years (b_6 = -0.04; 95%CI, -0.16 to 0.08), reaching a change point at 3.6 years (95%CI, 3.41 to 3.74), followed by rapid linear increases from ages 3.6 to 9 years (slope difference b_8 - b_6 = 1.54; 95%CI, 1.40 to 1.68). At age 9, the average BMI was 26.01 kg/m^2 (95%CI, 25.23 to 26.78).

© 2025 Liu C et al. *JAMA Network Open*.

eDiscussion. Limitations of Previous Models and Advantages of the Multiphase Latent Growth Mixture Model

The multiphase growth mixture model is well-suited for research where individual-specific timing of change points and phase transitions are key interests, such as estimating change points in child BMI growth in this study, and the onset of anxiety disorder in school-aged children. Compared to the traditionally used "visual inspection of change point" method,38 where researchers visually pinpoint the nadir of the BMI trajectory directly from observed data, the multiphase approach offers advantages.25 The visual inspection method can only estimate change points at ages where BMI measurements are available, and its accuracy depends largely on the number of BMI assessments and intervals between them, limiting the ability to freely estimate the nadir or change point.38 In contrast, the multiphase growth mixture model enables the estimated nadir or change point to occur between observed BMI assessment time points, providing greater precision in identifying phase transitions.

Another advantage of the current model is that estimated parameters, such as the individual-specific change points and rates of change, are easily interpretable. However, interpretating change point and slope estimates in typically used models like mixed-effects models with polynomial functions and traditional growth mixture modeling, is often problematic.25 Moreover, the generalizability of these models becomes questionable once they are increasingly tailored to specific data patterns. A related advantage of the current model is that it estimates the mean timing of change point and the variation in the timing, whereas mixed-effects models with polynomial functions only provide the estimate of the mean timing. With mean and variation estimates, we can further examine associations between change points and

© 2025 Liu C et al. *JAMA Network Open*.

predictors or outcomes, which may be difficult for previous models since individual-specific timing of the change point may not be directly estimated and needs to be derived first.

© 2025 Liu C et al. *JAMA Network Open*.
