# Supplementary material for: Early-Life Factors and Body Mass Index Trajectories Among Children in the ECHO Cohort
Source: JAMA Netw Open. 2025 May 22;8(5):e2511835. doi: 10.1001/jamanetworkopen.2025.11835 (PMC12100454; doi:10.1001/jamanetworkopen.2025.11835)
Supplement: Supplement 3. — Data Sharing Statement [file jamanetwopen-e2511835-s003.pdf]

Data Sharing Statement

Liu. Early-Life Factors and Body Mass Index Trajectories Among Children in the ECHO Cohort. *JAMA Netw Open*. Published May 22, 2025. doi:10.1001/jamanetworkopen.2025.11835

Data
Data available: Yes
Data types: Deidentified participant data
How to access data: Select de-identified data from the ECHO Program are available through NICHD's Data and Specimen Hub (DASH). Information on study data not available on DASH, such as some Indigenous datasets, can be found on the ECHO study DASH webpage.
When available: With publication

Supporting Documents
Document types: Statistical/analytic code
How to access documents: c.liu@wsu.edu
When available: With publication

Additional Information
Who can access the data: researchers whose proposed use of the data has been approved
Types of analyses: for any purpose
Mechanisms of data availability: after approval of a proposal, and with a signed data access agreement
